# Protected generation of dissipative Kerr solitons in supermodes of coupled optical microresonators

**DOI:** 10.1126/sciadv.abm6982

**Published:** 2022-04-01

**Authors:** Alexey Tikan, Aleksandr Tusnin, Johann Riemensberger, Mikhail Churaev, Xinru Ji, Kenichi Nicolas Komagata, Rui Ning Wang, Junqiu Liu, Tobias J. Kippenberg

**Affiliations:** Institute of Physics, Swiss Federal Institute of Technology Lausanne (EPFL), CH-1015 Lausanne, Switzerland.

## Abstract

A photonic dimer composed of two evanescently coupled high-*Q* microresonators is a fundamental element of multimode soliton lattices. It has demonstrated a variety of emergent nonlinear phenomena, including supermode soliton generation and soliton hopping. Here, we present another aspect of dissipative soliton generation in coupled resonators, revealing the advantages of this system over conventional single-resonator platforms. Namely, we show that the accessibility of solitons markedly varies for symmetric and antisymmetric supermode families. Linear measurements reveal that the coupling between transverse modes, giving rise to avoided mode crossings, can be substantially suppressed. We explain the origin of this phenomenon and show its influence on the dissipative Kerr soliton generation in lattices of coupled resonators of any type. Choosing an example of the topological Su-Schrieffer-Heeger model, we demonstrate how the edge state can be protected from the interaction with higher-order modes, allowing for the formation of topological Kerr solitons.

## Teaser

Optical solitons in coupled resonators is a possible future of microcombs, realized today by exploiting the mode coupling symmetry.

## INTRODUCTION

The analogy between molecules and coupled resonator systems has been discussed in various studies (*1*–*3*). Similar to the molecular energy surface noncrossings [first pointed out by von Neumann and Wigner (*4*) in the early years of molecular quantum mechanics], different eigenmode families of an optical resonator experience avoided mode crossings (AMXs), leading to distortions of initially smooth (i.e., unperturbed) dispersion profile (*5*–*8*). The dispersion profile is important in a plurality of resonant nonlinear wave-mixing schemes and especially in the context of dissipative Kerr soliton (DKS) generation in resonators having χ^(3)^ nonlinear susceptibility (*8*).

DKSs are localized stable structures found in driven-dissipative nonlinear resonators (*9*, *10*). The shape of DKS is given by the balance between dispersion and Kerr nonlinearity, while its amplitude is fixed owing to the balance between losses and parametric gain (*11*–*13*). The DKS generation in single optical microresonators (*10*) that can be integrated on a chip (*14*) has triggered the development of compact broadband frequency combs for various applications (*15*–*19*). Although AMX has been successfully used for triggering nonlinear dynamics in normal dispersion resonators (*20*–*23*), to control disorder (*24*), and to achieve a quiet point (*25*), it remains an undesirable effect, which disrupts the DKS formation process (*8*) and induces instabilities (*26*).

**Fig. 1.**
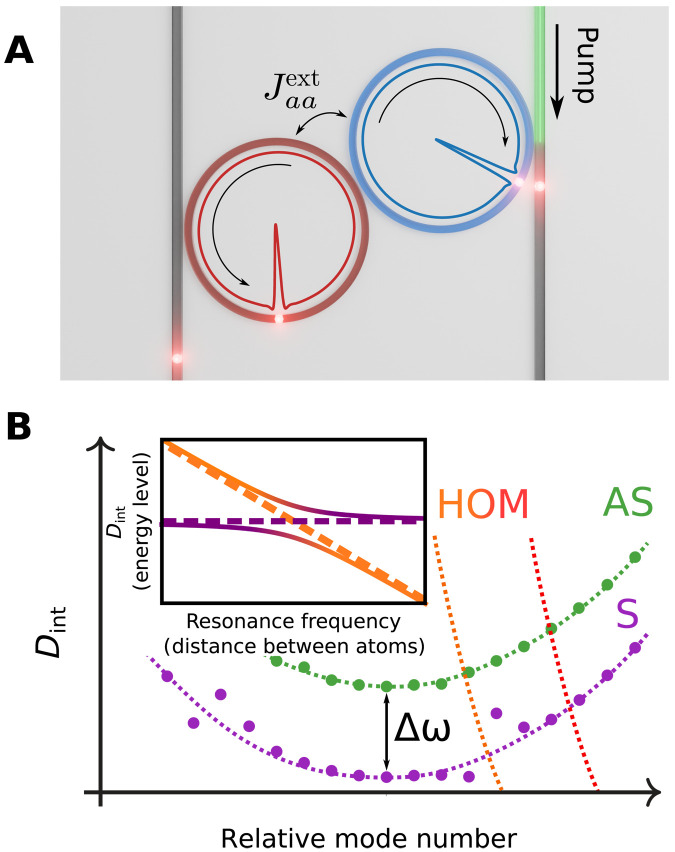
Mode interaction in a photonic dimer. (**A**) Schematic representation of the driven-dissipative photonic dimer with an interresonator coupling rate $J_{\mathrm{aa}}^{\text{ext}}$ and generated supermode DKS. (**B**) Schematic dispersion profile of the photonic dimer, showing the modal crossing structure. The inset shows avoided (solid line) and conical (dashed line) crossings in resonators and molecular systems. *D*_int_ stands for the integrated microresonator dispersion.

Recently, the possibility of the DKS generation in a photonic dimer (i.e., a pair of strongly coupled resonators as shown in Fig. 1A) has been investigated both experimentally and numerically (*27*, *28*). DKSs can be generated in one of the hybridized dimer supermodes called antisymmetric (AS) and symmetric (S) (see Fig. 1B). Theoretical studies have predicted a variety of emergent nonlinear phenomena including periodic appearance of commensurate and incommensurate dispersive waves, symmetry breaking, and soliton hopping (*27*). While AS supermode DKS generation has been readily achieved in experiments, some of the predicted effects (such as soliton hopping) were not observed experimentally because of an unexpectedly enhanced interaction of the S supermode family with higher-order modes (HOMs). The influence of AMX on the S supermodes strongly disrupts DKS generation and completely suppresses it above a certain power level. In contrast, the interaction of the AS modes with HOMs (see Fig. 1B) can be completely eliminated. This effect is ubiquitous in strongly coupled resonator arrangements.

In this work, we investigate the effect of protection against AMXs that are the primary obstacle for the experimental realization of recently introduced nested solitons in high-*Q* coupled resonator optical waveguides (*29*) and topological lattices (*30*). We provide an experimental study of the DKS generation in different supermodes of a photonic molecule realized with integrated Si_3_N_4_ microresonators (*31*), which confirms our observation. Investigating building blocks of the soliton lattices (broadly hybridized coupled trimer and plaquette of resonators), we prove the universal nature of this effect and demonstrate the DKS generation in the protected mode of the degenerate resonator plaquette. We propose a general model explaining the effect of protection and apply it to the topological Su-Schrieffer-Heeger (SSH) arrangement, demonstrating the failure of the topological protection. Furthermore, we provide a recipe for harnessing the effect for on-demand protection of the dispersion profile, which is essential for the experimental generation of edge state soliton frequency combs.

## RESULTS

### Photonic dimer

**Fig. 2.**
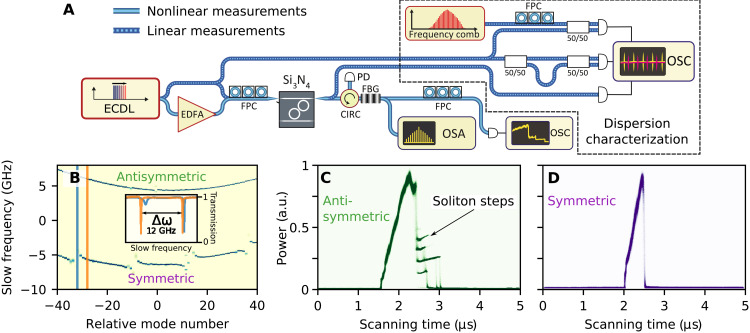
Effect of protection against mode crossings in the photonic dimer and supermode DKS generation. (**A**) Schematic representation of the experimental setup. ECDL, external cavity diode laser; EDFA, erbium-doped fiber amplifier; FPC, fiber polarization controller; CIRC, optical circulator; FBG, fiber Bragg grating; OSA, optical spectrum analyzer; OSC, sampling oscilloscope; PD, photodiode. ECDL is coupled into and out of the microresonator chip via lensed fibers. The dashed fiber path corresponds to the dispersion measurement scheme. (**B**) Dispersion profile measurements of the photonic dimer. The inset shows two cross sections of the plot at mode numbers −32 and −28 corresponding to AMX and its close vicinity, respectively. (**C** and **D**) Experimental recording of generated light in AS and S modes of the photonic dimer, respectively. Each plot contains 600 superimposed oscilloscope traces. The color density indicates a number of superimposed traces at a given point. a.u., arbitrary units.

Experimental evidences of the protection effect are obtained with strongly coupled microrings having ≈200 GHz free spectral range and loaded *Q* factor of the order of 2 million, realized on the Si_3_N_4_ platform using the photonic Damascene reflow process (*32*). The resonators are designed to feature an identical free spectral range. The intrinsic loss rate of the dimer is 50 MHz, and both resonators are interfaced with bus waveguides having external coupling rates of 100 MHz. Using frequency comb calibrated diode laser spectroscopy [see Fig. 2A and (*33*)], we retrieve first the dispersion profile of the photonic dimer. Figure 2B depicts the transmission spectrum of the photonic dimer in the form of an echellogram, where consecutive vertical lines are spaced by the free spectral range *D*_1_/2π of the microring resonator at the pump frequency ω_0_. The slow frequency denotes the frequency offset of the resonance from the equidistant grid and, therefore, reveals the integrated dispersion [*D*_int_(μ) = ω_μ_ − (ω_0_ + *D*_1_μ)] of the photonic dimer modes directly. The dispersion profile reveals two dimer supermode families. The fundamental S supermode family is strongly affected by AMXs, while the AS supermode dispersion profile is almost unperturbed. The inset of Fig. 2B shows a cross section of the plot at the mode crossed by a HOM (orange line) and before it (blue line). HOMs and, therefore, AMXs are likely to be present in soliton-generating microresonators because the waveguide constituting the microresonator is usually chosen to be multimode to guarantee low propagation losses (hence high *Q* factor) by reducing the influence of the fabrication-induced surface roughness (*34*).

We also study the influence of AMXs on the generation of supermode DKSs. Figure 2 (C and D) shows a superposition of 600 transmission traces obtained by sweeping the pump laser frequency over the AS and S resonances at 1554 nm with an optical power in the waveguide of 43 mW. We use a conventional continuous wave pumping scheme combined with fast single sideband tuning to eliminate thermal heating and resonance shifts (*35*). The strong pump line is filtered using fiber Bragg gratings, and the light generated by nonlinear processes in the resonator is sampled using a fast photodetector with 1-GHz bandwidth. Generated light profiles for the AS mode, detected with a photodiode after filtering out the pump comb line, systematically show the presence of characteristic steps signifying the stable access to the solitonic state (*10*, *36*). In contrast, S supermodes exhibit no solitonic feature at the equivalent pump power. At lower input powers, soliton generation can be observed in the S mode family; however, it depends on the particular distribution of AMXs on the dispersion profile. As pointed out in (*37*), the presence of the AMX leads to the intense generation of dispersive waves, which perturb the solitonic state. Each soliton acts as a source of dispersive waves and, therefore, the number of solitons is naturally reduced until the perturbation becomes sufficiently weak to maintain the state. The strength of the perturbation depends on the position of AMX because the power spectral density of the soliton and, hence, the optical power transferred to the HOM decay exponentially from the pumped mode.

Although the soliton generation has several key aspects including the quality factor of the resonators and stability of the pump source, we refer to this effect as protected generation of DKSs in supermodes, implying that the problems related to the soliton generation in a single resonator are addressed. Therefore, the key limitation in this case is the detuning rate between resonators that can only be controlled up to 70 to 80 GHz, resulting in the reduced (compared to the single resonator) fabrication yield.

### Photonic trimer and plaquette

**Fig. 3.**
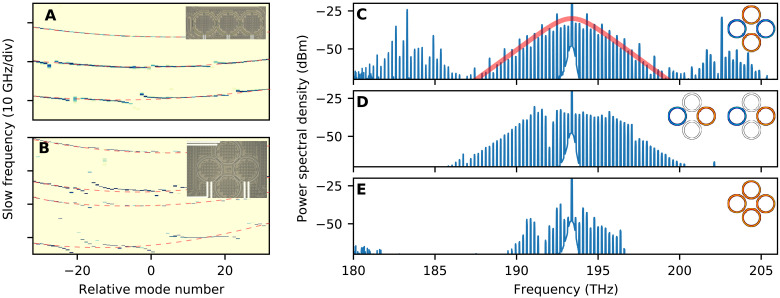
Effect of protection in photonic trimer and plaquette. (**A**) Linear dispersion measurement of a chain of three coupled resonators (photonic trimer). (**B**) The same for a square lattice (plaquette). Insets show microscope images of the Si_3_N_4_ microresonators of **≈**200 GHz free spectral range and imprinted system of heaters. (**C** to **E**) Optical spectra obtained by investigating the square lattice tuned into the degenerate state similar to the trimer configuration. Spectral density corresponds to top, middle, and bottom resonances of the effective trimer, respectively. The red line shows a fitting with the hyperbolic secant profile. Insets show a schematic representation of the supermode distribution.

A similar effect is observed in the trimer configuration. Linear dispersion measurements (see Fig. 3A) reveal that the protection effect is the strongest for the trimer supermode with the highest relative frequency and gradually decreases for lower frequency states on the integrated dispersion profile. We also investigate a more complex resonator arrangement representing a fundamental element of the square lattice—a plaquette. Figure 3B shows the corresponding dispersion profile. In the ideal case, two central mode families are degenerate. However, because of the presence of the finite interresonator detuning δ, the degeneracy is lifted, and we observe all four mode families. Imprinting metallic heaters on top of the device, we establish a control over the detuning of each individual resonator, thereby bringing the plaquette system to the degenerate state. We choose to work with this system as it represents a more general case of coupled resonators, including the trimer to the degenerate case. Figure 3 (C to E), corresponding to the upper-, middle (2× degenerate)-, and lower-frequency resonance, shows nonlinear probing of the plaquette structure. As follows from the figure, DKS in such configuration can be generated only in the upper resonance, which is expected to be protected from interaction with HOM.

The intersection of the soliton line with the dispersive parabolas in the nonlinear dispersion relation (*27*), as shown in fig. S1, results in the generation of dispersive waves that can be identified by the presence of strong sidebands in the multisoliton spectrum shown in Fig. 3C. Therefore, the low-noise radio frequency generation in systems of coupled resonators is an open question that is a subject of future investigations.

### Model of mode crossing suppression

To shed light on the protection phenomenon, we derive from Maxwell’s equations a Hermitian model of four coupled modes interaction (see the Supplementary Materials). We consider two fundamental *a*_1(2)_ and two transverse HOMs *b*_1(2)_ of both resonators constituting the dimer. The coupled mode equations can be written as follows (*38*)

$$i\frac{dU}{\mathrm{dt}}=-(\begin{array}{cccc} -\omega_{1} & J_{\mathrm{aa}}^{\text{ext}} & J_{\mathrm{ab}}^{\text{int}} & J_{\mathrm{ab}}^{\text{ext}}\\ J_{\mathrm{aa}}^{\text{ext}} & -\omega_{1} & J_{\mathrm{ab}}^{\text{ext}} & J_{\mathrm{ab}}^{\text{int}}\\ J_{\mathrm{ba}}^{\text{int}} & J_{\mathrm{ba}}^{\text{ext}} & -\omega_{2} & J_{\mathrm{bb}}^{\text{ext}}\\ J_{\mathrm{ba}}^{\text{ext}} & J_{\mathrm{ba}}^{\text{int}} & J_{\mathrm{bb}}^{\text{ext}} & -\omega_{2} \end{array})U$$ (1)

where **U** = (*a*_1_, *a*_2_, *b*_1_, *b*_2_)^⊤^. Eigenvalues of the coupling matrix can be found analytically. Assuming that the coupling matrix is symmetric, we obtain

$$\begin{array}{c} \lambda_{1,2}(\text{as})=\frac{1}{2}(2J_{\mathrm{aa}}^{\text{ext}}\pm \sqrt{4{\left(J_{\mathrm{ab}}^{\text{int}}-J_{\mathrm{ab}}^{\text{ext}}\right)}^{2}+{\left(\omega_{1}-\omega_{2}\right)}^{2}}+\omega_{1}+\omega_{2})\\ \lambda_{3,4}(s)=\frac{1}{2}(-2J_{\mathrm{aa}}^{\text{ext}}\pm \sqrt{4{\left(J_{\mathrm{ab}}^{\text{int}}+J_{\mathrm{ab}}^{\text{ext}}\right)}^{2}+{\left(\omega_{1}-\omega_{2}\right)}^{2}}+\omega_{1}+\omega_{2}) \end{array}$$ (2)

**Fig. 4.**
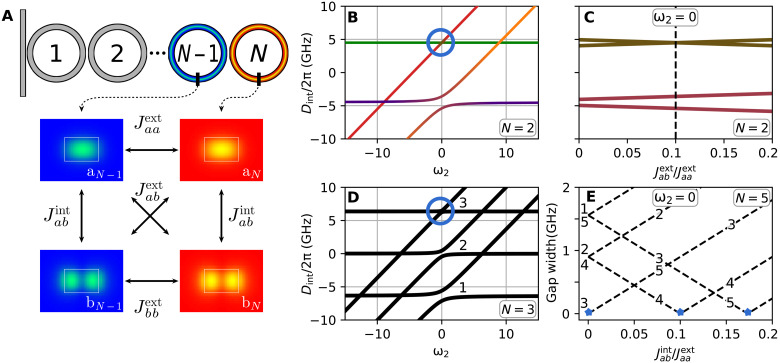
Effect of protection in coupled resonators. (**A**) Schematic representation of coupled resonator chain and description of the coefficients of the coupling matrix used in Eq. 1. (**B**) Protection in the photonic dimer. The protected state is highlighted by the blue circle. The green line corresponds to AS fundamental mode, the violet line corresponds to S fundamental mode, the red line corresponds to AS HOM, and the orange line corresponds to S HOM. Parameters are chosen to be close to the experimental ones: $J_{\mathrm{aa}}^{\text{ext}}/2\pi$ = 4.5 GHz, $J_{\mathrm{ab}}^{\text{int}}$ = $J_{\mathrm{ab}}^{\text{ext}}$ = 0.1$J_{\mathrm{aa}}^{\text{ext}}$. (**C**) The splitting between hybridized supermodes with coinciding central frequencies. The dashed black line shows parameters corresponding to the cross section of plot (B) at ω_2_ = 0. (**D**) Protection in the trimer configuration. (**E**) The gap distance between split resonances as a function of $J_{\mathrm{ab}}^{\text{int}}$/$J_{\mathrm{aa}}^{\text{ext}}$ for a chain of five coupled resonators at ω_2_ = 0, keeping the value of $J_{\mathrm{ab}}^{\text{ext}}/2\pi =0.1J_{\mathrm{aa}}^{\text{ext}}/2\pi$ = 0.45 GHz. Lines are numbered according to the relative frequency of the modes, as shown in plot (D). Blue stars depict the protected states.

The notation for coupling coefficients is described in Fig. 4A. $J_{\mathrm{aa}}^{\text{ext}}$ corresponds to the coupling between fundamental modes of the nearest resonators, $J_{\mathrm{ab}}^{\text{ext}}$ corresponds to the coupling between the fundamental mode of one resonator and the HOM of the neighbor, and $J_{\mathrm{ab}}^{\text{int}}$ is the coupling between fundamental and HOM within the same resonator. The coupling strength between two HOMs is set to $J_{\mathrm{aa}}^{\text{ext}}$ because it does not qualitatively change the result. The difference between $J_{\mathrm{aa}}^{\text{ext}}$ and $J_{\mathrm{bb}}^{\text{ext}}$ leads to a shift of the hybridization area along the direction of the HOM. As an example of the resonator HOM, we show Transverse electric mode (TE_10_). The influence of the dissipative coupling in our system is negligible (*39*) and, therefore, is not included in the analysis.

Thus, we find two pairs of eigenvalues that represent the mode interaction. The first couple λ_1,2_ corresponds to the AS supermodes, while λ_3,4_ corresponds to the S one. The expression under the square root in the first couple of eigenvalues contains the term ${\left(J_{\mathrm{ab}}^{\text{int}}-J_{\mathrm{ab}}^{\text{ext}}\right)}^{2}$. Therefore, in the case when $J_{\mathrm{ab}}^{\text{int}}$ and $J_{\mathrm{ab}}^{\text{ext}}$ are of the same order, the influence of the AMX is reduced. However, in the second couple of eigenvalues, the effect of AMX is increased in comparison to the conventional hybridization in the single resonator case.

Numerical simulations of the coupling region for parameters close to experimental ones (see the Supplementary Materials) demonstrated that the ratio between $J_{\mathrm{ab}}^{\text{ext}}$ and $J_{\mathrm{ab}}^{\text{int}}$ tends to 1 for silicon nitride–based anomalous dispersion ring microresonators. The coupling sections to bus and drop waveguides will contribute to the coefficient $J_{\mathrm{ab}}^{\text{int}}$ as well; however, this contribution is found to be one order of magnitude smaller, which is consistent with the experimentally observed strong protection of the AS supermode parabola (Fig. 2B).

The eigenvalue system, Eq. 2, as a function of the central frequency of the HOM ω_2_ with the ratio $J_{\mathrm{ab}}^{\text{int}}/J_{\mathrm{ab}}^{\text{ext}}=1$ is depicted in Fig. 4B. Figure 4C shows the dependence of the hybridized mode position for ω_1_ = ω_2_ (at the center of Fig. 4B) as a function of $J_{\mathrm{ab}}^{\text{ext}}$/$J_{\mathrm{aa}}^{\text{ext}}$. Black dashed lines correspond to the conditions considered in Fig. 4B. As predicted from Eq. 2, when $J_{\mathrm{ab}}^{\text{int}}$ and $J_{\mathrm{ab}}^{\text{ext}}$ coincide exactly, the gap distance tends to 0.

The structure of the coupling matrix in Eq. 1 is notably similar to the Hamiltonian discussed in (*40*), which underpins the profound nature of the analogy with molecular systems. Similar effects, known as conical or diabolical crossings (*41*) in this community, have been actively investigated at the end of the last century.

This model can be easily extended to the case of arbitrary number of resonators (see the Supplementary Materials). An example of the mode hybridization for the trimer configurations is shown in Fig. 4D. Influence of the AMX increases with the decreasing relative frequency, as suggested by the experimental data. Numerical analysis of longer chains revealed that the index of the protected mode depends on the values $J_{\mathrm{ab}}^{\text{ext}}$ and $J_{\mathrm{ab}}^{\text{int}}$ and, therefore, can be manipulated. When $J_{\mathrm{ab}}^{\text{ext}}$ can be neglected, then the effect of AMXs is the same for all the hybridized modes. In the opposite case, when $J_{\mathrm{ab}}^{\text{int}}\ll J_{\mathrm{ab}}^{\text{ext}}$, the protection falls into the middle mode family and symmetrically decreases toward modes with higher and lower relative frequencies. Therefore, the protection can be moved along the dispersion relation by changing the coupling coefficients ratio. Figure 4E shows the dependence of the AMX-induced gap width as a function of normalized $J_{\mathrm{ab}}^{\text{int}}$ coefficient for a five resonator chain computed using the same parameters as previously. When $J_{\mathrm{ab}}^{\text{int}}$ can be neglected, the middle mode (3) becomes protected. With increasing $J_{\mathrm{ab}}^{\text{int}}$, the protection moves toward the fourth mode family and subsequently to the fifth one. Although there are a number of techniques allowing one to suppress the excitation of HOMs in a microresonator (*42*–*44*), this can lead to the reduction of the coefficient $J_{\mathrm{ab}}^{\text{int}}$ while the control of both $J_{\mathrm{ab}}^{\text{int}}$ and $J_{\mathrm{ab}}^{\text{ext}}$ is required.

### Protection of topological states in the SSH model

**Fig. 5.**
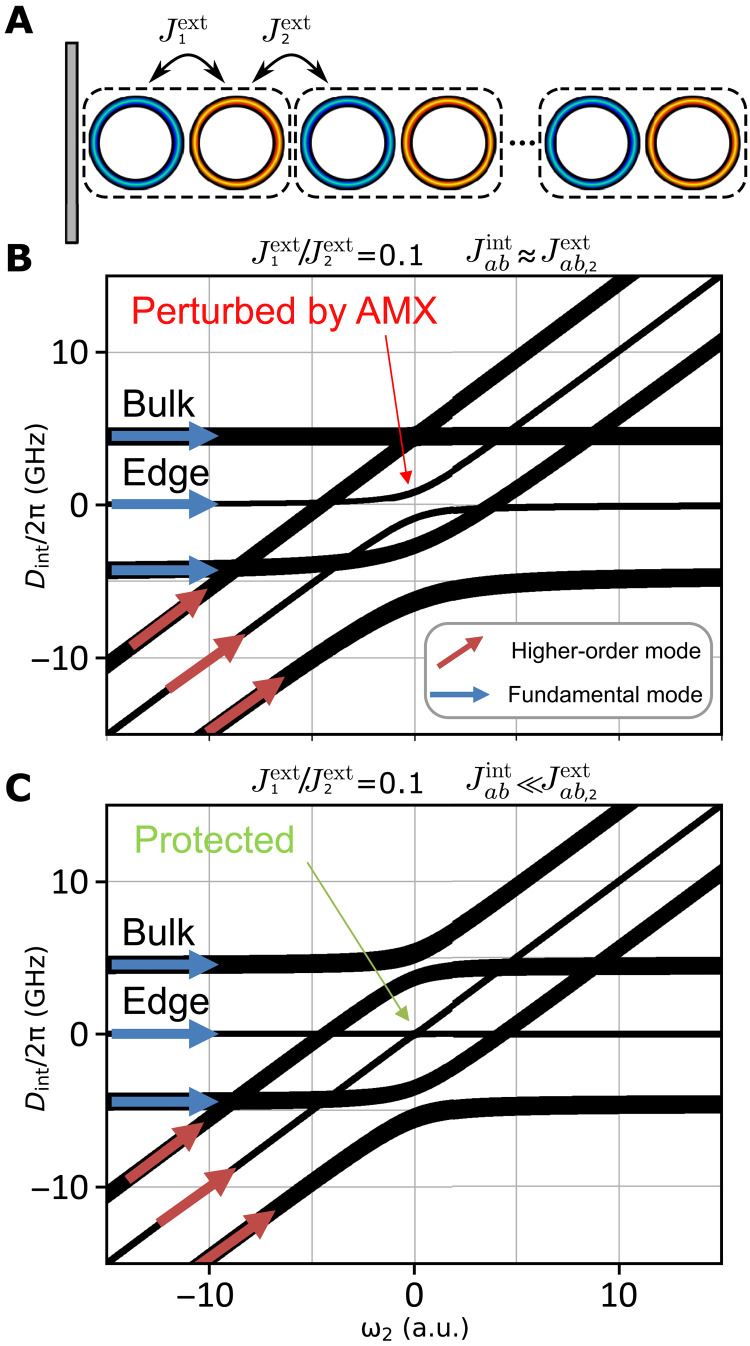
Protection against mode crossing of the topological edge state in SSH model. (**A**) Schematic representation of 10 coupled resonators with alternating coupling, which constitutes the SSH chain. (**B**) Influence of AMX when $J_{\mathrm{ab}}^{\text{int}}\approx J_{\mathrm{ab},2}^{\text{ext}}$. (**C**) The same configuration with $J_{\mathrm{ab}}^{\text{int}}\ll J_{\mathrm{ab},2}^{\text{ext}}$ ($J_{\mathrm{ab}}^{\text{int}}=0.001J_{\mathrm{ab},2}^{\text{ext}}$) exhibits the protection against AMX.

To highlight the importance of protection for the future development of the field of the soliton lattices, we study the effect of AMXs on topologically protected edge states in the SSH model (*45*) originally proposed for the explanation of mobile neutral defects in polyacetylene. This model is described by the following Hamiltonian: ${\hat{H}}_{\text{SSH}}=\sum_{n}t_{1}{\hat{c}}_{n}^{†}{\hat{c}}_{n-1}+t_{2}{\hat{c}}_{n+1}^{†}{\hat{c}}_{n}$, where ${\hat{c}}_{n}^{†}$ is the creation operator at the *n*th site and *t*_1,2_ is the hopping amplitudes. Because of the simplicity of implementation (*46*), this model often serves as a primary verification platform for nonlinearity-related topological effects (*47*). It can be realized in our system by varying the interresonator coupling coefficients $J_{\mathrm{aa}}^{\text{ext}}$ and $J_{\mathrm{ab}}^{\text{ext}}$ playing the role of the hopping amplitudes here. Schematic representation of the SSH chain is shown in Fig. 5A. The alternating coupling effectively divides the chain into a number of unit cells shown by dashed rectangles. The coupling strength ratio inside a unit cell and between unit cells ($t_{1}/t_{2}=J_{\mathrm{aa},\mathrm{ab},1}^{\text{ext}}/J_{\mathrm{aa},\mathrm{ab},2}^{\text{ext}}$) is chosen to be 0.1, which is sufficient for opening a wide photonic bandgap and obtaining a nonzero integer winding number—a topological property, invariant under adiabatic perturbations (*48*).

Figure 5B shows the mode hybridization for the SSH model realized with 10 resonators. The topologically protected (against the disorder and variation of the coupling coefficients) edge state is in the middle of the gap between two bulk states as shown in Fig. 5B. The same is observed for the HOM family. According to the model, a crucial influence of AMX on the topological state is expected, which potentially forbids or markedly obstructs the generation of topological DKSs if the protection is not controlled. AMXs in this system, together with interresonator detuning in absolute frequency, act as on-side potential breaking the chiral symmetry of the SSH lattice (*49*) and omitting the inherent topological protection (*50*). However, increasing the difference between $J_{\mathrm{ab}}^{\text{int}}$ and $J_{\mathrm{ab}}^{\text{ext}}$, we observe enhancement of the protection effect on the edge state modes. This can be achieved by carefully designing the coupling section to suppress the internal transverse mode couplings. Choosing the ratio $J_{\mathrm{ab}}^{\text{int}}=0.001J_{\mathrm{ab},2}^{\text{ext}}$, we observe a complete protection against AMX as shown in Fig. 5C.

## DISCUSSION

In conclusion, we introduced the notion of protection against AMX in chains of coupled multimode resonators, which exhibit a remarkable similarity with conical energy level crossings in molecular systems. The crucial influence of this effect on the dispersion profile and, therefore, the supermode DKS generation is demonstrated experimentally. We propose a simple model that fully explains the effect and proposes a way to harness it for stable DKS generation. We highlight its importance by showing that the topologically protected edge state in the SSH model can be highly influenced by AMXs if the mode interaction is not carefully controlled and, therefore, it must be taken into the account while designing the experimental platform for the observation of topological solitons.

## MATERIALS AND METHODS

### Device fabrication

Silicon nitride photonic devices are fabricated at Center of MicroNanoTechnology (CMi), EPFL using the photonic Damascene reflow process, deep ultraviolet stepper lithography, and silica preform reflow method. Silicon nitride is a complementary metal-oxide semiconductor–compatible material, having Kerr nonlinearity and good power-handling capability. The resonators are designed to have similar free spectral range *D*_1_/2π ≈ 200 GHz, which corresponds to 125-μm radius. The chosen waveguide dimensions are 800 nm (height) and 1.5 μm (width), resulting into the group velocity dispersion coefficient *D*_2_/2π = 4.1 MHz. In the dimer and plaquette case, all resonators are interfaced with waveguides to monitor the optical field in the resonators, while in the trimer case, only the first and the last ones are out-coupled. We optimize the input and output coupling to the device by designing double-inverse tapers.

### Dispersion characterization

The linear dispersion measurements are provided with a frequency comb–calibrated diode laser spectroscopy technique. In this way, we are able to extract the information about the width of each resonance and its offset from the equidistant grid. The linear parameters are extracted by the fitting of the recorded data by using the coupled-mode theory and thereby extracting the information about the internal loss rate, the coupling rate to the bus and drop waveguides, and the integrated dispersion. To extend the spectral range of the dispersion measurements, we use two tunable external cavity diode lasers (ECDLs) (Santec, TSL-550) with overlapping spectral ranges (1500 to 1630 nm and 1350 to 1505 nm). Each laser scan takes about 20 s.

### Experimental setup

The experimental setup for the characterization of nonlinear effects in the coupled optical microresonators comprises a widely tunable ECDL operating around the C-band and amplified by an erbium-doped fiber amplifier, a polarization controller, filtering fiber Bragg gratings, a pair of high-precision control stages, and an adjustable chip holder. We inject the pump to the photonic chip using lensed fibers. The pump laser is tuned into antisymmetric resonance with the fast single-sideband tuning, which requires an arbitrary electrical signal generator, a voltage controlled oscillator, and an in-phase and quadrature (IQ) modulator.

The generated signal is retrieved either at the bus or at the drop waveguides. To suppress the strong pump light, we sequentially use two fiber Bragg gratings. The reflected signal is redirected by an optical circulator and sent to a power controlling photodiode. The transmitted signal is recorded by a fast photodiode and sent to an oscilloscope. Optical spectra generated by the nonlinear interaction in the presented photonic molecules are analyzed using an optical spectrum analyzer (Yokogawa, AQ6370).
