# Supplementary material for: Protected generation of dissipative Kerr solitons in supermodes of coupled optical microresonators
Source: Sci Adv. 2022 Apr 1;8(13):eabm6982. doi: 10.1126/sciadv.abm6982 (PMC10938571; doi:10.1126/sciadv.abm6982)
Supplement: Supplementary file 1 — Supplementary Text Figs. S1 to S4 [file sciadv.abm6982_sm.pdf]

Science Advances
AAAS

Supplementary Materials for

Protected generation of dissipative Kerr solitons in supermodes of coupled optical microresonators

Alexey Tikan*, Aleksandr Tusnin, Johann Riemensberger, Mikhail Churaev, Xinru Ji, Kenichi Nicolas Komagata, Rui Ning Wang, Junqiu Liu, Tobias J. Kippenberg*

*Corresponding author. Email: alexey.tikan@epfl.ch (A.Ti.); tobias.kippenberg@epfl.ch (T.J.K.)

Published 1 April 2022, *Sci. Adv.* **8**, eabm6982 (2022)
DOI: 10.1126/sciadv.abm6982

This PDF file includes:

Supplementary Text
Figs. S1 to S4

NUMERICAL SIMULATIONS OF SOLITON GENERATION IN A DEGENERATE PHOTONIC PLAQUETTE

In order to support the experimental data and justify the recorded shape of the soliotonic spectrum of the photon, we perform numerical simulations of four coupled Lugiato-Lefever equations. We use step-adaptative Dormand-Prince Runge-Kutta method of Order 8(5,3) with numerical boxes each having 512 points. The simulation parameters are chosen be similar to the experimental ones: integrated group velocity dispersion D_2=10 MHz, inter-resonator coupling J=4.5 GHz, 1st (pumped) and 3rd rings are coupled to a waveguide with the rate κ_{ex}=70 MHz, Si_3N_4 waveguide has dimensions 1.5x0.8 μm, free spectral range is 181 GHz. The parameters are identical for every ring constituting the plaquette. Solitons were excited by tuning over the resonance to the soliton existence region and further hard-seeded with a fixed detuning. The pump (0.8 W) is injected to the first ring only. The integration time is 1 μ seconds. The nonlinear dynamics over the slow time is sampled with 1000 points.

SFig. 1 (a) shows an averaged optical spectrum, of the field generated in the pumped resonator. The spectrum demonstrates features similar to the experimentally recorded one. There is a symmetric enhancement of ≈55th mode optical power which, taking in the account the free spectral ranges of the resonators, corresponds to ≈10 THz. This is in a good agreement with the reported experimental observations. The symmetric enhancement of comb lines signifies the presence of strong dispersive waves in the cavity. The corresponding intracavity temporal waveform is shown in SFig. 1 (b). The main (sech-shaped) part of the spectrum has a periodic perturbation similar to the one recorded experimentally. This perturbation is caused by the presence of multiple (two in this case) solitons in the cavity. The dispersive waves appear as a periodic perturbation of the background as well as the solitons. This makes soliotns oscillating with time as shown in the spatio-temporal diagram for the intracavity power profile. Using the spatio-temporal diagram, we can reconstruct the nonlinear dispersion relation by applying a double Fourier transform. The nonlinear dispersion relation reveals the origin of the of comb enhancement. The soliton line (since the dispersion is compensated by the nonlinearity) crosses the dispersion parabola which corresponds to another supermode of the system. The crossing point is shown with white circles.

ADDITIONAL EXPERIMENTAL DATA

In order to demonstrate that the effect of protection is not linked to a particular device, we present here a set of linear measurements. Fig.2 suggests that we observe a similar behavior in a number of devices.

Further, we present additional data for the soliton generation in the photonic plaquette. Namely, simultaneously with the optical spectra, we recorded the transmission trace. The presence of distinct steps in the transmission trace is one of the key signatures of the dissipative Kerr soliton generation in microresonators. The traces were generated by using a fast single-sideband tuning technique. As follows from SFig. 3, only the resonance having higher absolute frequency (tuning is done from the blue-detuned to the red-detuned side of the resonance) demonstrates the presence of steps.

MODE INTERACTIONS IN TWO EVANESCENTLY COUPLED RESONATORS

We examine here the mode interaction of two ring resonators using a perturbation approach. Taking the solution of the Maxwell's equations for a single resonator as a basis for the field profile in the coupled system, we derive coupling coefficients between different modes as a function of mode overlaps. The obtained expressions can be used for the exact evaluation of the coupling coefficients for whispering gallery mode resonators, since analytical expressions are known in this case, while for integrated microresonator dimers they, however, help to qualitatively understand the mode-crossing mechanism.

We start from the scalar wave equation on electric field in the system comprising two identical evanescently coupled optical resonators. The wave equation

$$(\Delta + \frac{n_g^2}{c^2}\frac{\partial^2}{\partial t^2})E = 0 \quad \text{(S1)}$$

governs the electric field in the media with the group index n_g. In a single resonator case, rotational symme-

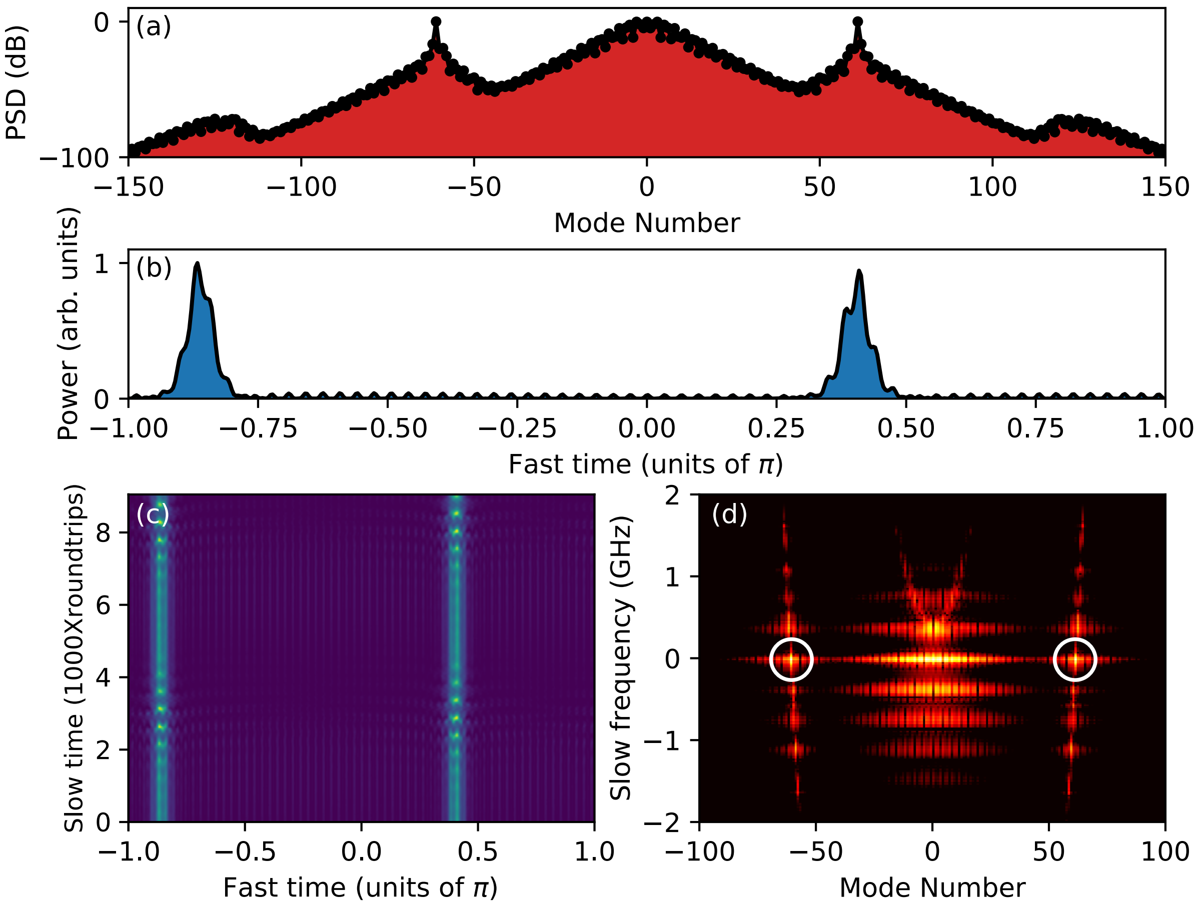

Supplementary Figure 1. **Solitons in a phonic plaquette.** All data is shown for the pumped resonator. (a) Power spectra density (PSD) of two solitons averaged over 0.5 μsec. (b) Temporal profile of dissipative Kerr solitons (DKSs). The horizontal axis depicts the inter-resonator azimuth angle $\in$(-π, π). (c) Spatio-temporal diagram. (d) Corresponding nonlinear dispersion relation. White circles show crossing points of the soliton line with the lower parabola.

try allows one to obtain a set of eigenfrequencies and eigenfunctions supported by the system. Typically, there are two polarization mode families (TE and TM), and within each polarization mode family there is a set of eigenfunctions (i.e. states) which have different spatial distributions. In the ideal case, all the eigenfunctions are orthogonal, even if they correspond to degenerate eigenfrequencies. The presence of perturbations which cause the axial symmetry breaking leads to the interaction between the modes breaking, thereby their orthogonality. This interaction manifest itself as avoided mode crossings (AMXs) in the dispersion profile. The AMXs appear at degenerate frequencies, where two different modes have close eigenvalues.

The scenario of mode interaction in the photonic dimer is similar to the conventional single resonator case, but the difference arises from the fact that we investigate two sets of eigenmodes which belong to different rings. In order to obtain the coupling coefficients, we employ the perturbation approach. Starting from independent eigenfunctions for both rings, implying at first the infinite distance between the resonators, we decompose the electric field on a series of eigenfunctions and obtain a system of coupled ordinary differential equations on amplitudes of the modes.

We start with a singe resonator case which has the group index n_{g_I}. Using the ansatz of harmonic time dependence $E \rightarrow Ee^{-i\omega t}$, one obtains the Helmholtz equation

$$(\Delta + n_{g_I}^2 k_0^2)E = 0, \tag{S2}$$

where $k_0 = \omega/c$ is the wavenumber. Eq. (S2) defines eigenfrequencies ω_μ^I and eigenfunctions Ψ_μ^I with orthogonality relation:

$$\int \Psi_\mu^I (\Psi_\nu^I)^* n_{g_I}^2 dV = \delta_{\mu,\nu}. \tag{S3}$$

Here asterisk stands for complex conjugation. The electric field is $E = A_\mu^I \Psi_\mu^I$, where A_μ^I is the normalization constant. Note, the same is valid for the second resonator, for which it is sufficient simply to replace I by

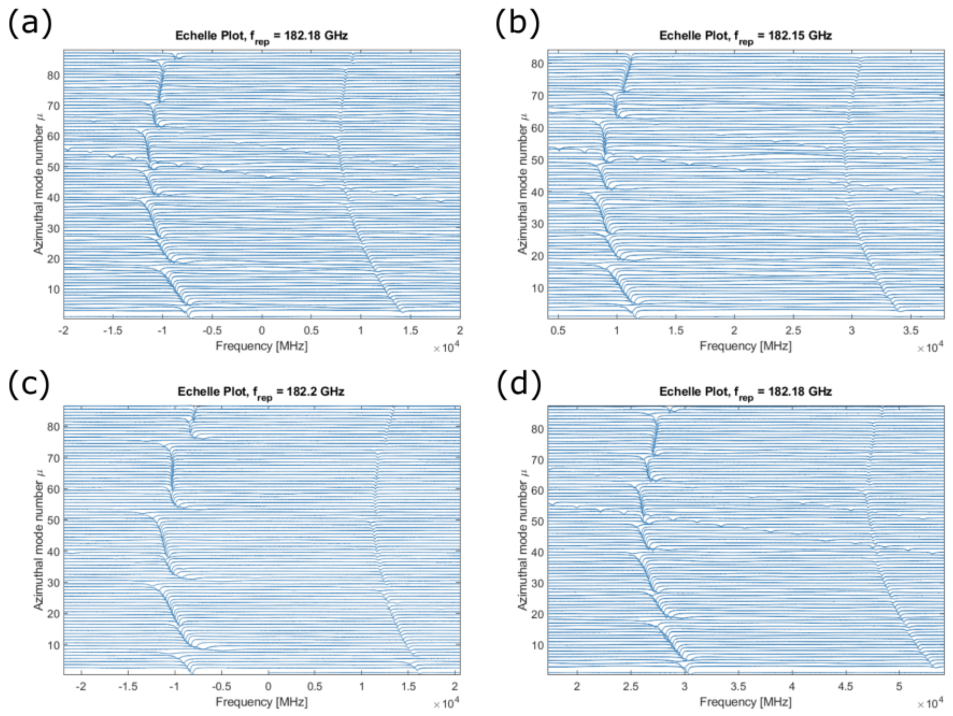

Supplementary Figure 2. Linear spectroscopy of different photonic dimers. All the devices have similar parameters and fabricated on the same wafer.

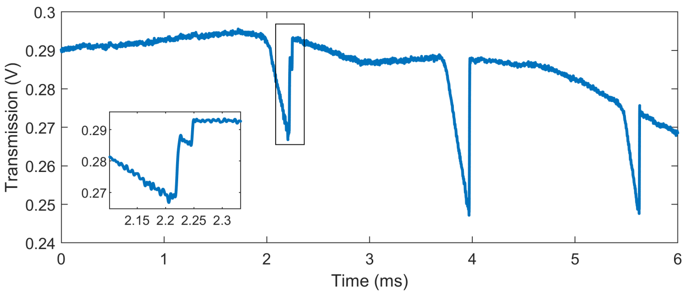

Supplementary Figure 3. Filtered transmission trace. The case of a phonic plaquette. The resonance on the left demonstrates the presence of soliton steps. The pump laser detuning is swept from higher to lower frequencies.

II. Moreover, we consider the resonators to be identical, meaning that $\omega_\nu^I = \omega_\nu^{II}$.

Now, we suppose that the two resonators are placed closed so their eigenfunctions overlap. In order to exploit the eigenfuctions of each ring, we rewrite the group index n_g in the following form:

$$n_g^2 = \begin{cases} n_{g_I}^2 + n_{II} \\ n_{g_{II}}^2 + n_I, \end{cases} \tag{S4}$$

depending on the basis we want to use. We decompose further the electric field in the following way:

$$E = \sum_i A_i^I(t)\Psi_i^I e^{-i\omega_i t} + \sum_i A_i^{II}(t)\Psi_i^{II} e^{-i\omega_i t}. \tag{S5}$$

Substituting this to the Eq. S1 and using the slowly varying envelope approximation ($d^2A_i^I/dt^2 \ll \omega_i dA^I/dt$) we obtain the following equation:

$$\Delta E - \frac{n_g^2}{c^2}\frac{\partial^2 E}{\partial t^2} =$$
$$\sum_i \left(n_{II}\Psi_i^I A_i^I \lambda_i^2 + 2i\lambda_i \dot{A}_i^I \Psi_i^I \frac{(n_{g_I}^2 + n_{II})}{c} \right) e^{-i\omega_i t} +$$
$$\sum_i \left(n_I \Psi_i^{II} A_i^{II} \lambda_i^2 + 2i\lambda_i \dot{A}_i^{II} \Psi_i^{II} \frac{(n_{g_{II}}^2 + n_I)}{c} \right) e^{-i\omega_i t} = 0, \tag{S6}$$

where $\lambda_i = \omega_i/c$ and $\dot{A}$ stands for the time derivative of A. Now, we multiply this equation by $(\Psi_k^{I(II)})^*$ and integrate it over the whole volume. Using the orthogonality relation (S3), one obtains a system of ordinary differential equations on the mode amplitudes with coupling coefficients proportional to the mode overlap.

Considering the case of two mode families in both resonators, one can derive the matrix model introduced in the main text. In order to keep the same notations, we denote $A_1^I \equiv a_1$,$A_2^I \equiv b_1$, $A_1^{II} \equiv a_2$, $A_2^{II} \equiv b_2$. Taking only leading order coefficients, system (S6) takes form

$$\begin{cases} \dot{a}_1 &= i(J_{a_1a_1}a_1 + J_{a_1a_2}a_2 + J_{a_1b_1}b_1 + J_{a_1b_2}b_2) \\ \dot{a}_2 &= i(J_{a_2a_1}a_1 + J_{a_2a_2}a_2 + J_{a_2b_1}b_1 + J_{a_2b_2}b_2) \\ \dot{b}_1 &= i(J_{b_1a_1}a_1 + J_{b_1a_2}a_2 + J_{b_1b_1}b_1 + J_{b_1b_2}b_2) \\ \dot{b}_2 &= i(J_{b_2a_1}a_1 + J_{b_2a_2}a_2 + J_{b_2b_1}b_1 + J_{b_2b_2}b_2), \end{cases} \tag{S7}$$

where diagonal terms indicate self-frequency shift due to presence of the coupling sections. They can be expressed through the mode overlap integrals as follows:

$$J_{a_1a_1} = \frac{\lambda_0 c}{2}\int \Psi_0^I \Psi_0^{I^*} n_{II} dV; \; J_{a_2a_2} = \frac{\lambda_0 c}{2}\int \Psi_0^{II} \Psi_0^{II^*} n_I dV \tag{S8}$$

$$J_{b_1b_1} = \frac{\lambda_1 c}{2}\int \Psi_1^I \Psi_1^{I^*} n_{II} dV; \; J_{b_2b_2} = \frac{\lambda_1 c}{2}\int \Psi_1^{II} \Psi_1^{II^*} n_I dV. \tag{S9}$$

Due to the symmetry, the expressions in each line of Eq. (S8) are equal. Applying the notations from the main text, we obtain $\omega_1 = J_{a_1a_1}$ and $\omega_2 = J_{b_1b_1}$.

The offdiagonal coefficients in system (S7) depict the mode interaction. Let us consider the interaction between the fundamental and higher order modes of one resonator. The corresponding coefficients are expressed as

$$J_{a_1b_1} = \frac{\lambda_1^2 c}{2\lambda_0}\int \Psi_1^I \Psi_0^{I^*} n_{II} dV e^{-i(\omega_1-\omega_0)t}, \tag{S10}$$

$$J_{a_2b_2} = \frac{\lambda_1^2 c}{2\lambda_0}\int \Psi_1^{II} \Psi_0^{II^*} n_{I} dV e^{-i(\omega_1-\omega_0)t}, \tag{S11}$$

$$J_{b_1a_1} = \frac{\lambda_0^2 c}{2\lambda_1}\int \Psi_0^I \Psi_1^{I^*} n_{II} dV e^{-i(\omega_0-\omega_1)t}, \tag{S12}$$

$$J_{b_2a_2} = \frac{\lambda_0^2 c}{2\lambda_1}\int \Psi_0^{II} \Psi_1^{II^*} n_{I} dV e^{-i(\omega_0-\omega_1)t}. \tag{S13}$$

As one can see, the interaction efficiency is enhanced at the points of degeneracy, where the eigenfrequencies coincide. These points correspond to the exact positions of the mode crossings. In the main text we consider this particular example, thus $J_{ab}^{\text{int}} = J_{a_1b_1}|_{\omega_0=\omega_1} = J_{a_2b_2}|_{\omega_0=\omega_1}$ and $J_{ba}^{\text{int}} = J_{b_1a_1}|_{\omega_0=\omega_1} = J_{b_2a_2}|_{\omega_0=\omega_1}$, where we assumed the coupling purely real for simplicity.

The coupling coefficients between the fundamental modes of both resonators take form:

$$J_{a_1a_2} = \frac{\lambda_0 c}{2}\int \Psi_0^{II} \Psi_0^{I^*} n_I dV, \tag{S14}$$

$$J_{a_2a_1} = \frac{\lambda_0 c}{2}\int \Psi_0^{I} \Psi_0^{II^*} n_{II} dV, \tag{S15}$$

and they are equal due to the symmetry. The corresponding coefficient in the main text $J_{aa}^{\text{ext}} = J_{a_1a_2} J_{a_2a_1}$. In the similar way, we express coupling between higher order modes

$$J_{b_1b_2} = \frac{\lambda_1 c}{2}\int \Psi_1^{II} \Psi_1^{I^*} n_I dV, \tag{S16}$$

$$J_{b_2b_1} = \frac{\lambda_1 c}{2}\int \Psi_1^{I} \Psi_1^{II^*} n_{II} dV, \tag{S17}$$

with $J_{bb}^{\text{ext}} = J_{b_1b_2} = J_{b_2b_1}$.

The coefficients governing interactions between fundamental and higher order modes of distinct resonators are placed on the side diagonal of the system (S7), and their expressions are by

$$J_{a_1b_2} = \frac{\lambda_1^2 c}{2\lambda_0}\int \Psi_1^{II} \Psi_0^{I^*} n_{I} dV e^{-i(\omega_1-\omega_0)t}, \tag{S18}$$

$$J_{a_2b_1} = \frac{\lambda_1^2 c}{2\lambda_0}\int \Psi_1^{I} \Psi_0^{II^*} n_{II} dV e^{-i(\omega_1-\omega_0)t}, \tag{S19}$$

$$J_{b_2a_1} = \frac{\lambda_0^2 c}{2\lambda_1}\int \Psi_0^{I} \Psi_1^{II^*} n_{II} dV e^{-i(\omega_0-\omega_1)t}, \tag{S20}$$

$$J_{b_1a_2} = \frac{\lambda_0^2 c}{2\lambda_1}\int \Psi_0^{II} \Psi_1^{I^*} n_{I} dV e^{-i(\omega_0-\omega_1)t}. \tag{S21}$$

As one can see, the interaction increases at degenerate frequencies, and then $J_{ab}^{ext} = J_{a_1b_2}|_{\omega_1=\omega_2} = J_{a_2b_1}|_{\omega_1=\omega_2}$, $J_{ba}^{ext} = J_{b_2a_1}|_{\omega_1=\omega_2} = J_{b_1a_2}|_{\omega_1=\omega_2}$. It is important to note, that the intraresonator interaction originates from the mode overlap in the area where both modes decay exponentially (for example see Eq. (S10)), when the interesonator coupling originates from the area where one is localized and second one is evanescent (e.g. Eq. (S18)). However, it is hard to estimate the ratio between these coefficients because it also depends on the integral along azimuth coordinate. In order to obtain this ratio, we provide FDTD simulations, which are presented in the next sections.

MATRIX MODEL

Generalized to the case of N coupled resonators, the coupling matrix of the size 2Nx2N can be represented as follows:

$$i\frac{dU}{dt} = -\begin{pmatrix}
-\omega_1 & J_{aa}^{ext} & & & J_{ab}^{int} & J_{ab}^{ext} & & \\
J_{aa}^{ext} & -\omega_1 & \ddots & & J_{ab}^{ext} & J_{ab}^{int} & \ddots & \\
 & \ddots & \ddots & J_{aa}^{ext} & & \ddots & \ddots & J_{ab}^{ext} \\
 & & J_{aa}^{ext} & -\omega_1 & & & J_{ab}^{ext} & J_{ab}^{int} \\
J_{ba}^{int} & J_{ba}^{ext} & & & -\omega_2 & J_{bb}^{ext} & & \\
J_{ba}^{ext} & J_{ba}^{int} & \ddots & & J_{bb}^{ext} & -\omega_2 & \ddots & \\
 & \ddots & \ddots & J_{ba}^{ext} & & \ddots & \ddots & J_{bb}^{ext} \\
 & & J_{ba}^{ext} & J_{ba}^{int} & & & J_{bb}^{ext} & -\omega_2
\end{pmatrix} U, \tag{S22}$$

where $U = (a_1, ..., a_N, b_1, ..., b_N)^\intercal$. The coupling matrix is comprised of four blocks of symmetric tridiagonal matrices, implying that empty spaces are zeros. For the calculations presented in the article we suppose that $J_{ab}^{ext,int}$ and $J_{ab}^{ext,int}$ are equal due to the apparent summery, therefore second and third blocks of the coupling matrix are identical. Blocks one and four are also set to be identical since the difference between J_{aa}^{ext} and J_{bb}^{ext} will lead to a simple shift along the direction of the higher-order mode and the mode interaction therefore has to be examined at $\omega_1 = \omega_2 = 0$.

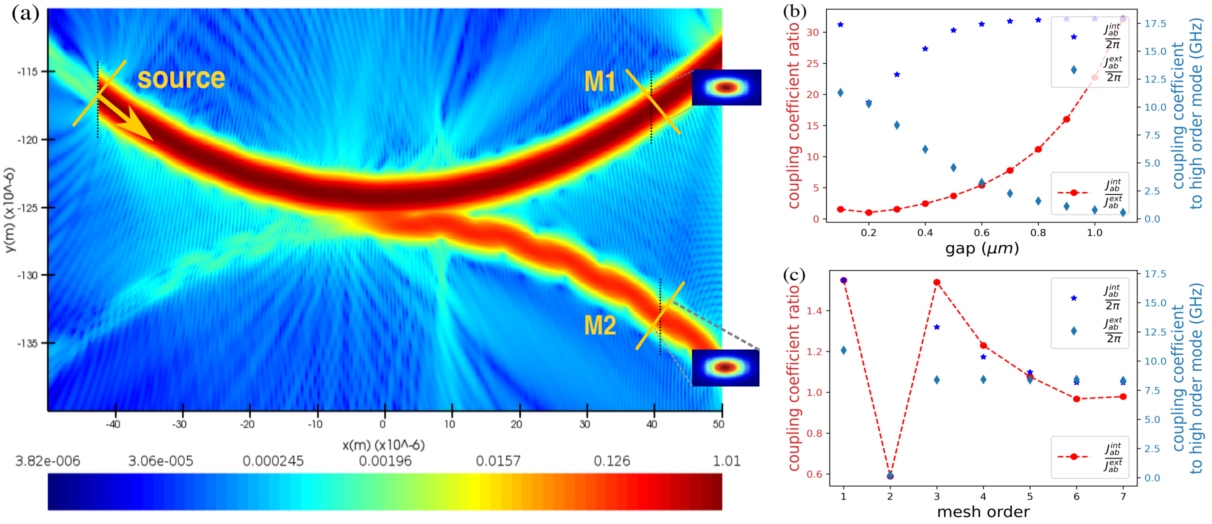

Supplementary Figure 4. FDTD simulations of the coupling section. (a) Normalized electric field distribution shown in log scale, the colour bar measures the field power. The mode source is launched normal to the waveguide propagation direction with unity power. Insets present spatial field distributions recorded by monitor M1 and M2. The gap distance is set as 0.3 μm. (b) Dependence of the coupling coefficients to higher-order modes on gap distance, at mesh 3. (c) Convergence of the coupling coefficients to higher-order modes with mesh order, as illustrated by the red dashed line, the coupling coefficient ratio converges closely to unity.

FDTD SIMULATIONS OF THE COUPLING SECTION

In order to confirm the coupling coefficient ratio expected from the analytical model of four-mode interaction, we provide FDTD simulations of the coupling section of the photonic dimer. We constructed a model of a dimer device comprised of two 200 GHz ring resonators. The silicon nitride (Si_3N_4) resonator core is fully cladded with Silicon dioxide (SiO_2). Both resonators are 1.5 μm wide and 0.82 μm high, with sidewall angle $\alpha = 90°$, as used in the experiments. The mode source was configured to inject at an angle of 20° with unity power, as shown in SFig. 4a, and to excite only the fundamental mode of the ring. In this way, we shrank the simulation region to 100 × 30 × 8 μm^3 and the simulation time to 900 fs, which is sufficient to capture correctly the coupling to higher-order modes with much less processing time. The boundary of the simulation region is fixed with a perfectly matched layer (PML) condition to absorb the incident light and therefore to prevent backreflection. The light field then propagated in the full simulation region until a stationary state is reached. Monitors M0, M1 and M2 recorded the spatial distributions of the mode source, the transmitted field and the coupled field respectively. In addition, two mode expansion monitors were placed in the same plane as M1 and M2 to calculate the power of selected resonator eigenmode (TE_{10}). All powers are normalized as they derived from the resonator fundamental mode that is launched with unity power. The coupling coefficients, J_{ab}^{int} and J_{ab}^{ext}, are estimated using a simplified coupled mode equation, by $J_{ab}^{i} = D_1 \times \arccos(\sqrt{1 - P_{ab}^{i}})$, where $D_1/2\pi = FSR$.

Numerical simulations reveal that increasing the gap distance between two resonators, J_{ab}^{ext} rapidly (eventually exponentially) decays, while J_{ab}^{int} remains constant at 18 GHz except the region 0.2-0.4 μm where it demonstrates lower values at mesh order 3 as shown in SFig. 4. Careful analysis of the ratio $J_{ab}^{int}/J_{ab}^{ext}$ convergence with increasing mesh order (decreasing the simulation net size) suggests that the ratio converges to unity. The simulations with mesh order 3 gives a considerable error of $\approx 35\%$.

The coupling of the ring resonator to the bus and drop waveguides was also simulated at 0.3 μm, rendering a converged result of 0.75 GHz, which contributes to the coefficient $J_{ab}^{\rm int}$.
